# Sulphur availability modulates *Arabidopsis thaliana* responses to iron deficiency

**DOI:** 10.1371/journal.pone.0237998

**Published:** 2020-08-20

**Authors:** Kevin Robe, Fei Gao, Pauline Bonillo, Nicolas Tissot, Frédéric Gaymard, Pierre Fourcroy, Esther Izquierdo, Christian Dubos

**Affiliations:** BPMP, Univ Montpellier, CNRS, INRAE, Institut Agro, Montpellier, France; Iwate University, JAPAN

## Abstract

Among the mineral nutrients that are required for plant metabolism, iron (Fe) and sulphur (S) play a central role as both elements are needed for the activity of several proteins involved in essential cellular processes. A combination of physiological, biochemical and molecular approaches was employed to investigate how S availability influences plant response to Fe deficiency, using the model plant *Arabidopsis thaliana*. We first observed that chlorosis symptom induced by Fe deficiency was less pronounced when S availability was scarce. We thus found that S deficiency inhibited the Fe deficiency induced expression of several genes associated with the maintenance of Fe homeostasis. This includes structural genes involved in Fe uptake (i.e. *IRT1*, *FRO2*, *PDR9*, *NRAMP1*) and transport (i.e. *FRD3*, *NAS4*) as well as a subset of their upstream regulators, namely *BTS*, *PYE* and the four clade Ib bHLH. Last, we found that the over accumulation of manganese (Mn) in response to Fe shortage was reduced under combined Fe and S deficiencies. These data suggest that S deficiency inhibits the Fe deficiency dependent induction of the Fe uptake machinery. This in turn limits the transport into the root and the plant body of potentially toxic divalent cations such as Mn and Zn, thus limiting the deleterious effect of Fe deprivation.

## Introduction

Plant growth and development depends on both the availability of mineral nutrients present in the soil and the capacity of the root system to mine the rhizosphere in search for these nutrients. Therefore, the ability of plants to acquire and assimilate mineral nutrients impinges on crop productivity and the quality of their derived products [1].

Among the mineral nutrients required for plant metabolism, iron (Fe) and sulphur (S) play a central role as both are needed for the activity of several proteins involved in essential cellular processes (e.g. photosynthesis, respiration, biosynthesis of primary and secondary metabolites). Within proteins, Fe and S are found as individual atoms or tightly connected to each other in the form of prosthetic groups, the Fe-S clusters. In plants, Fe-S proteins are mainly involved in electron transfer reactions (e.g. within the photosynthetic and respiratory electron transport chains in chloroplasts and mitochondria, respectively) or in the catalysis of oxidation-reduction reactions [2–5]. The biosynthesis of cysteine also connects Fe and S homeostasis, since the desulphuration of cysteine to alanine is a reaction in the assembly process of Fe-S clusters [2]. The biosynthesis of methionine, a sulphur-containing amino acid, is another hub that links Fe and S homeostasis. For instance, methionine is the precursor of molecules that play key roles during the plant response to Fe shortage, such as ethylene, nicotianamine (NA) or phytosiderophores (PS). In non-graminaceous species (strategy I plants for Fe acquisition), ethylene and NA are respectively involved in the activation of the transcriptional regulatory cascade controlling the response to Fe deficiency and the transport of Fe throughout the plant [6–8]. In graminaceous species (strategy II plants for Fe acquisition), PS, which derives from NA, are secreted into the rhizosphere in order to chelate and solubilize Fe and thus improve plant Fe nutrition [8]. Considering that Fe shortage arises in one third of the cultivated land at the surface of the planet and that the frequency of soils displaying S deficiency is increasing (mostly due to a decrease of anthropogenic S release; [9]), it became obvious that there is a need for understanding how Fe and S homeostasis regulation are interconnected in plants. This knowledge being necessary if one aims at sustaining plant growth and productivity in such limited environment with limited use of exogenous fertilizers.

This tight connection between Fe and S, at the molecular and physiological levels, as exemplified earlier through the Fe-S cluster biogenesis and methionine biosynthesis, suggested that coordination in the control of both nutrients homeostasis occurred. Indeed, several examples in the literature confirmed this assumption, whether the plants belong to eudicot or monocot clades. For instance, in tomato (*Solanum lycopersicum* L.) or rapeseed (*Brassica napus* L.), the Fe deficiency induced expression and activity of key genes associated with Fe acquisition was inhibited when plants were submitted to S deprivation [10, 11]. Similarly, the capacity of barley (*Hordeum vulgare* L.) seedlings grown under S deficiency to cope with Fe shortage was restored when S was provided [12]. Conversely, it was also demonstrated, in tomato and durum wheat (*Triticum turgidum* L. subsp. *durum*), that Fe shortage triggers responses associated with S deficiency [13–15]. In *Arabidopsis thaliana*, such cross talk between Fe and S homeostasis was also reported, notably through the study of the expression of the main Fe and S transporter present at the root epidermis, namely *IRT1* (*IRON-REGULATED TRANSPORTER 1*) and *SULTR1;1* (*SULPHATE TRANSPORTER 1;1*), respectively [16]. As expected, these studies also highlighted that Fe and S deficiency impact the expression of a common set of genes that are not directly related to Fe and S acquisition and assimilation, which was in adequacy with the central role that play both nutrients in several key metabolic processes [17–20].

The intricate connection between Fe and S homeostasis suggests that complex regulatory mechanism are at play. For instance, if the regulatory mechanisms controlling Fe and S homeostasis are quite well described [21–23], it remains to determine the means by which the information on the availability of one nutrient is integrated into the regulatory pathway controlling the homeostasis of the other one. The nature of the signals controlling this mechanism is also matter of debates. Because Fe and S are required in large amounts in chloroplasts and mitochondria, in particular in the form of Fe-S clusters, it has been proposed that one or more retrograde signal(s) deriving from these organelles might feedback to the nucleus to regulate the homeostasis of both nutrient [18, 24–27]. Among these potential signals, the SAL1/FRY1 (FIERY)-PAP chloroplast retrograde pathway emerged as an interesting candidate [24, 28]. For instance, PAP (3'-phosphoadenosine 5'- phosphate), an intermediate compound into the assimilation of S, plays a central role in plant response to several environmental stresses (e.g. osmotic, cold, drought, high light or response to pathogens) including nutrient availability [29–32]. Interestingly, it was also reported that SAL1-PAP retrograde signalling pathway was also involved in the maintenance of Fe homeostasis [33].

In this study we have investigated, using the model plant *Arabidopsis thaliana*, how S deficiency affects at the physiological and molecular levels the plant response to Fe shortage and how this signal is integrated into the transcriptional regulatory cascade controlling Fe deficiency responses. We first observed that chlorosis symptom induced by Fe deficiency was less pronounced when S availability was scarce. Because Fe deficiency is mostly regulated at the transcriptional level, we have investigated the expression of key genes involved in Fe homeostasis, including transcription factors (TFs). Expression analyses revealed that the Fe deficiency induced expression of most of the assayed genes was reduced when both Fe and S were limiting. Nevertheless, only a subset of TFs followed this expression pattern whereas the expression of the other set was unaffected by the double deficiency. Using loss-of-function mutants we found that the SAL1-PAP retrograde signalling pathway is most probably not involved in this process. The analysis of rosette leaves and roots micronutrients content highlighted that the increased accumulation of manganese (Mn) in response of Fe shortage was reduced under the combined Fe and S deficiencies. Altogether these data suggest that S deficiency, by inhibiting the Fe acquisition machinery that is induced in response to Fe deficiency, limits the unspecific transport into the root and the plant body of potentially toxic divalent cations, particularly Mn, thus limiting the deleterious effect of Fe deprivation.

## Results

### Iron deficiency induced chlorosis in *Arabidopsis thaliana* is less pronounced when sulphur availability is scarce

One of the most obvious symptoms that plants display when experiencing iron (Fe) deficiency is the yellowing of the leaf tissues. This phenomenon, also called chlorosis, is due to an altered accumulation of chlorophylls present in the interveinal tissues. In order to investigate how sulphur (S) availability may affect the plant response to Fe deficiency, we assessed how contrasting Fe and S nutrition regimes impact chlorophylls accumulation. For this purpose, three-week-old *Arabidopsis thaliana* plants were subjected for 10 days to Fe deficiency (-Fe +S), S deficiency (+Fe -S) and Fe and S deficiencies (-Fe -S), and compared to plants grown in control condition (+Fe +S).

**Fig 1:**
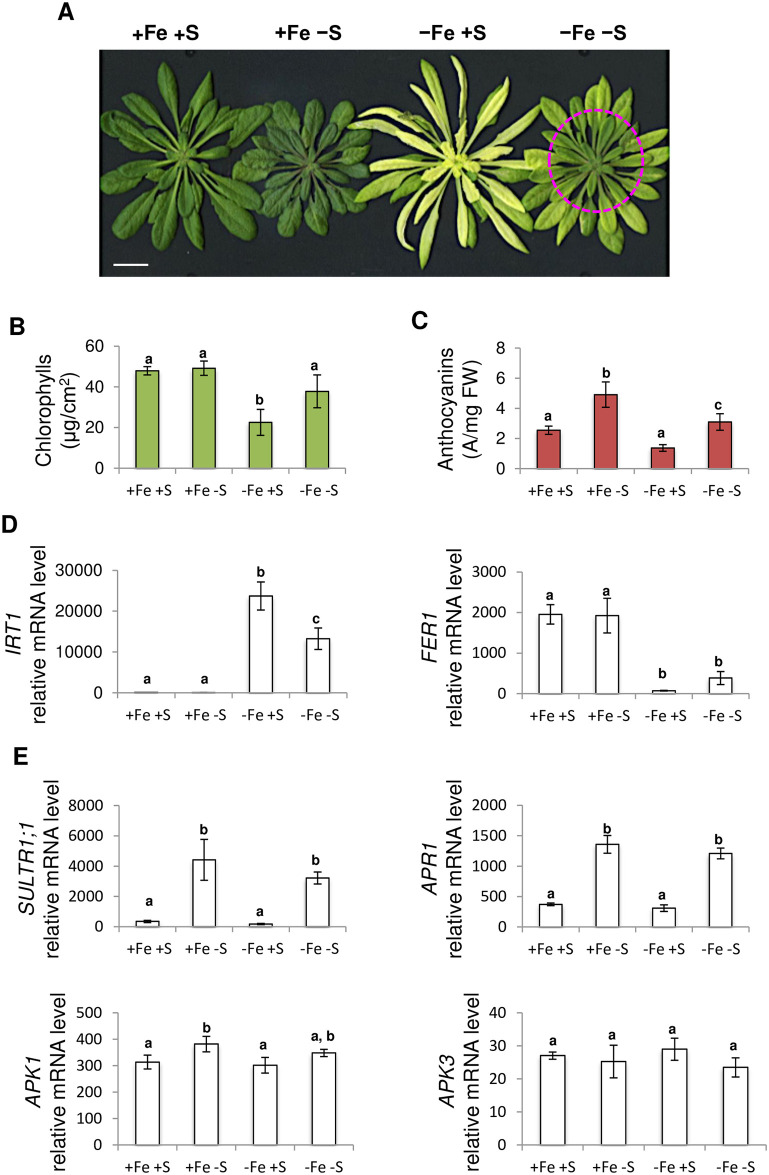
*Arabidopsis thaliana* iron deficiency induced chlorosis is less pronounced when sulphur availability is scarce. **(A)** Rosette phenotype of Arabidopsis plants grown for three weeks in presence of 25 μM Fe(III)-EDTA and then transferred for 10 days in four different media: control (+Fe +S), S deficiency (+Fe -S), Fe deficiency (-Fe +S) and Fe and S deficiencies (-Fe -S). Magenta arrows indicate the youngest leaves that formed during the 10 days of treatments. Bar = 1 cm. **(B)** Chlorophylls and **(C)** anthocyanins content of the youngest leaves of the rosette presented panel A (Magenta arrows). **(D)** Quantitative RT-PCR analysis of *IRT1* (-Fe marker) and *FER1* (+Fe marker) mRNA levels in plant roots following 5 days of treatment. **(E)** Quantitative RT-PCR analysis of *SULTR1;1* (-S marker), *APR1*, *APK1* and *APK3* mRNA levels in plant roots following 5 days of treatment. **(B-E)** Means with the same letter are not significantly different according to one-way ANOVA followed by post-hoc Tukey test (*P*< 0.05). n = 3 biological repeats from one representative experiment. Each experiment was repeated three times.

From the visual screening of the plants (Fig 1A), no striking differences were observed between plants grown in control and S deficiency conditions whereas, as expected, plants grown in Fe deficiency condition displayed a clear chlorosis phenotype. Strikingly, we observed that the chlorosis phenotype observed in plants grown under Fe deprivation was less pronounced when S availability was scarce (-Fe +S *vs* -Fe -S), in particular for the youngest leaves that developed during the course of the treatments. This observation was confirmed by measuring the chlorophylls content present in the young leaves of the plants grown in these four different conditions (Fig 1B). Anthocyanins content, which increases in tissues of plants facing S deficiency [34], was evaluated in order to assess the effect of S deficiency in our experiments. As expected, we found that anthocyanins accumulation was higher in plants submitted to S deficiency (+Fe -S and -Fe -S) when compared to plants grown in control or Fe deficiency conditions (Fig 1C).

At the molecular level, *IRT1* (*IRON-REGULATED TRANSPORTER 1*) expression, which encodes the high affinity Fe transporter present at the root epidermis, was induced in response to Fe deficiency and this induction was decreased in plants facing both Fe and S deficiencies (Fig 1D), in agreement with [16]. Steady state mRNA level of *FER1* (*FERRITIN 1*), which encodes a protein involved in the transient storage of Fe and whose expression is induced under excess Fe, was also measured. *FER1* expression decreased in plants grown under Fe deficiency, independently of the amount of S present in the medium (Fig 1D). *SULTR1;1* (*SULPHATE TRANSPORTER 1;1*) expression, which encodes the high affinity sulphate transporter present at the root epidermis, was induced in response to S deficiency, still in agreement with [16] (Fig 1E). The expression of *APR1* (*APS REDUCTASE 1*), *APK1* (*APS KINASE 1*) and *APK3* (*APS KINASE 3*), was also assayed as the encoded proteins play key roles in the partitioning of the assimilated S, in the form of APS (adenosine 5’-phosphosulfate), between the biosynthesis of cysteine (APR1), and thus methionine, and the formation of several secondary metabolites such as glucosinolates (APK1 and APK3). The expression of *APR1*, and to a lesser extent *APK1*, was similar to that one of *SULTR1;1*, whereas the expression of *APK3* remained unaffected by the treatments (Fig 1E).

**Fig 2:**
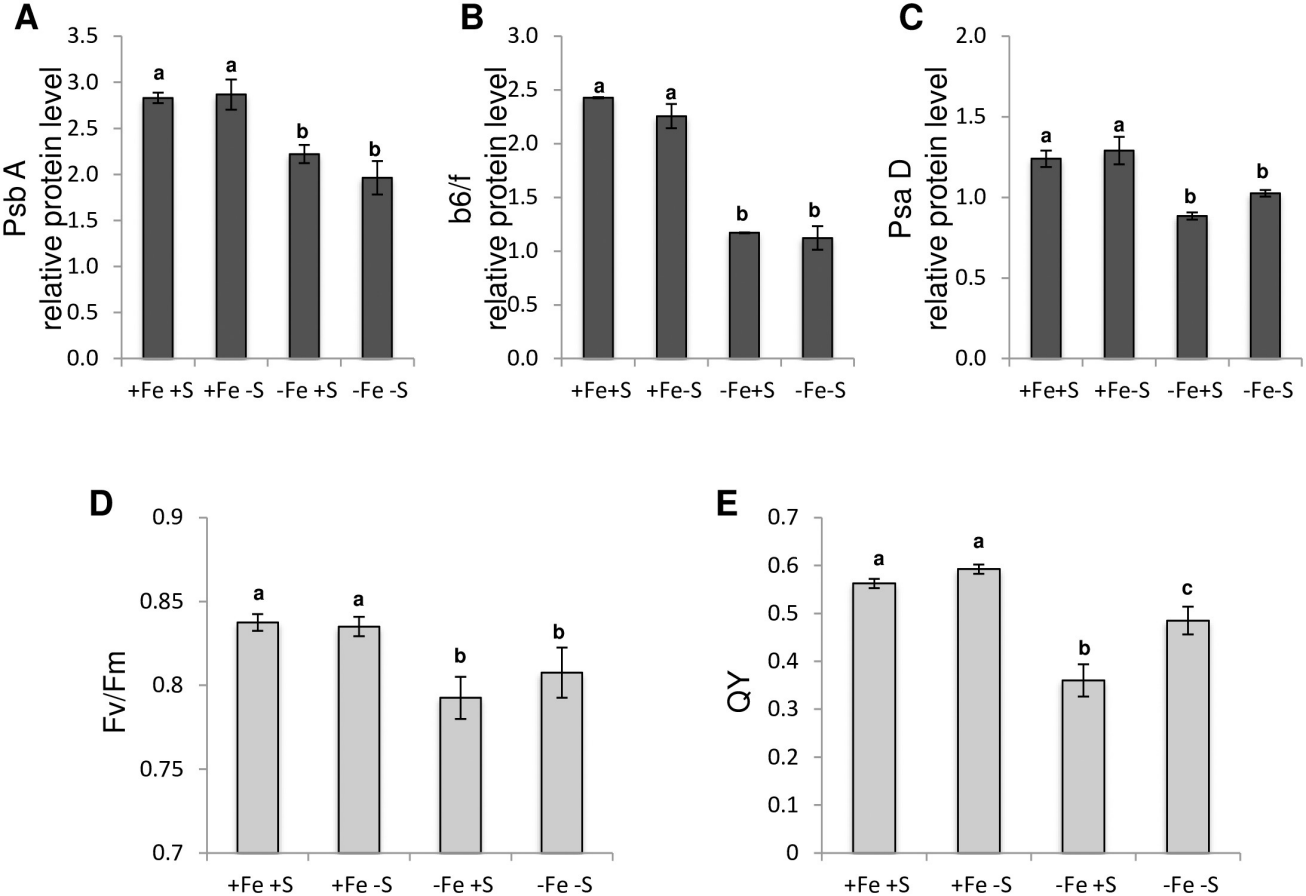
Fe deficiency effect on *Arabidopsis thaliana* rosette leaves photosynthetic activity is less pronounced when S availability is scarce. **(A-C)** Western blot analyses using total protein extracts from whole rosette leaves of Arabidopsis plants grown for three weeks in presence of 25 μM Fe(III)-EDTA and then transferred for 10 days in four different media: control (+Fe +S), S deficiency (+Fe -S), Fe deficiency (-Fe +S) and Fe and S deficiencies (-Fe -S). Three proteins related to the photosynthetic light reactions were analysed: **(A)** PsbA (photosystem II), **(B)** b6f (cytochrome b6f complex) and **(C)** PsaD (photosystem I). **(D-E)** Chlorophyll fluorescence parameters: **(D)** Fv/Fm of dark-adapted plants and **(E)** quantum yield of photochemical energy conversion. **(A-E)** Means with the same letter are not significantly different according to one-way ANOVA followed by post-hoc Tukey test (*P*< 0.05). n = 3 biological repeats from one representative experiment. Each experiment was repeated three times.

We then investigated how Fe and S deficiencies were affecting parameters associated with plant photosynthetic activity. We first analysed the accumulation of key proteins involved in the photosynthetic chain, namely the PHOTOSYSTEM II SUBUNIT A (PsbA), the CYTOCHROME b6/f COMPLEX SUBUNIT (b6/f) and the PHOTOSYSTEM I SUBUNIT D (PsaD). We confirmed that under Fe deficiency the three proteins accumulated less than in Fe sufficiency, in a manner that was independent of S availability (Fig 2A–2C) [35]. In order to get some insights into how the treatments affected the photosynthetic capacities of the plants, we focused our analysis on the youngest leaves, the ones that developed during the course of the treatments. For this purpose, we have measured the maximum quantum yield of PSII (F_v_/F_m_), which reflects the potential efficiency of PSII (Fig 2D), and the quantum yield (QY), that reflects the electron flux that goes through the photosynthetic chain (Fig 2E). F_v_/F_m_ and QY were unchanged in S deficiency when compared with control condition whereas both were significantly diminished by Fe deficiency. In contrast to F_v_/F_m_, QY was significantly higher in Fe and S dual deficiency than that in Fe deficiency alone.

### Sulphur deficiency inhibits the induction of the expression, in response to iron deficiency, of key Arabidopsis genes involved in the maintenance of iron homeostasis

**Fig 3:**
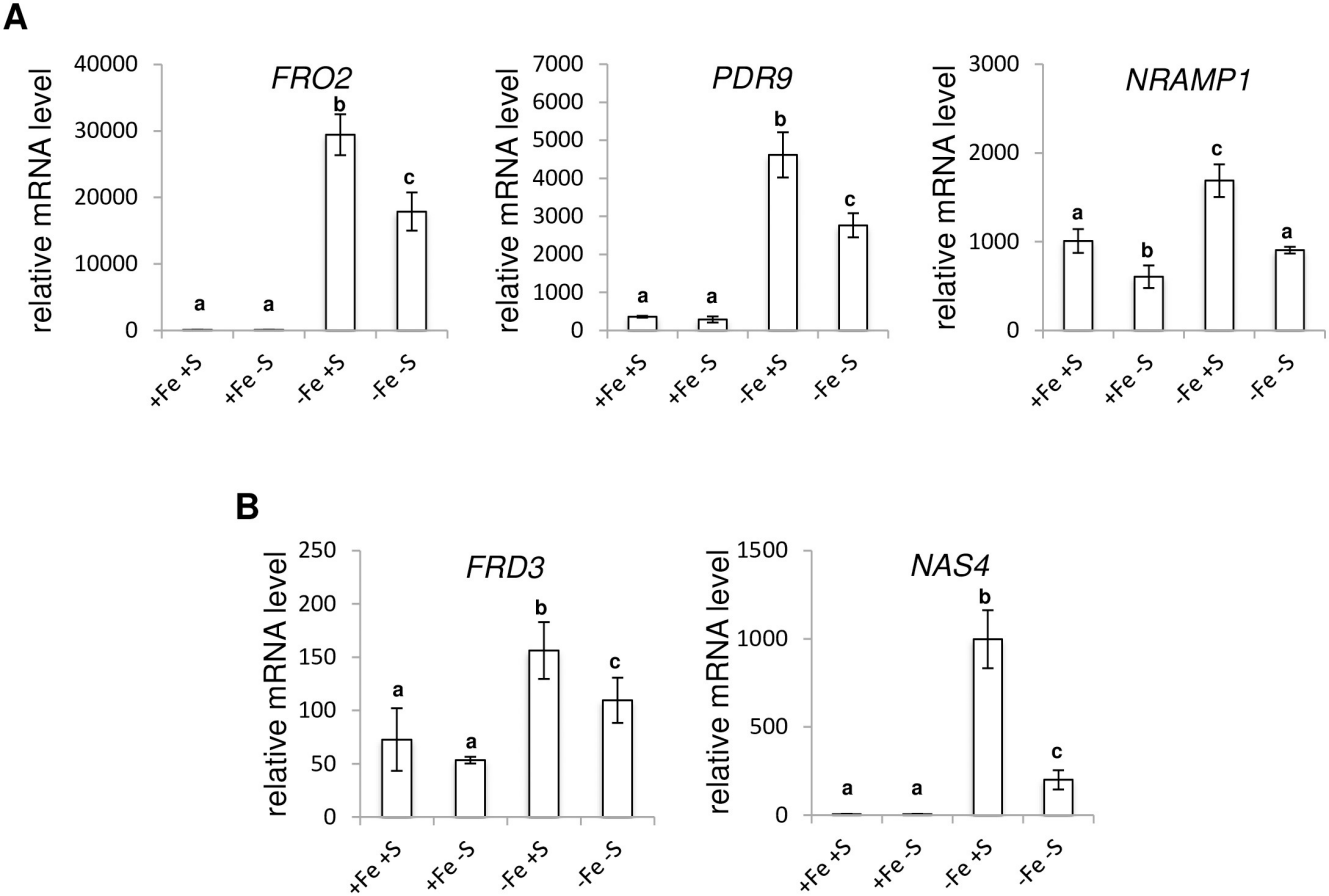
Expression analysis of genes involved in Fe uptake and transport in *Arabidopsis thaliana*. cDNA were synthesized from RNA extracted from roots of Arabidopsis plants grown for three weeks in presence of 25 μM Fe(III)-EDTA and transferred to four different media: control (+Fe +S), S deficiency (+Fe -S), Fe deficiency (-Fe +S) and Fe and S deficiencies (-Fe -S). Root samples were harvested five days after plants were transferred to the assayed media. Quantitative RT-PCR analyses were carried out on genes involved in **(A)** Fe uptake (*FRO2*, *PDR9* and *NRAMP1*) and **(B)** Fe transport (*FRD3* and *NAS4*). **(A-B)** Means with the same letter are not significantly different according to one-way ANOVA followed by post-hoc Tukey test (*P*< 0.05) n = 3 biological repeats from one representative experiment. Each experiment was repeated three times.

The expression of the high affinity Fe transporter *IRT1* in response to Fe deficiency (-Fe +S) is inhibited by S deficiency (-Fe -S) whereas S deficiency alone (+Fe -S) does not significantly affect its expression when compared to control (+Fe +S) condition (Fig 1D). Whether the expression of two other main genes involved in Fe acquisition, namely *FRO2* (*FERRIC REDUCTION OXIDASE 2*) and *PDR9* (*PLEIOTROPIC DRUG RESISTANCE 9/ABCG37*) [36], follow a similar expression pattern remained to be determined. We found that the expression pattern of both genes was comparable to that of *IRT1* (Fig 3A). This was also the case for the expression of the low affinity Fe transporter *NRAMP1* (*NATURAL RESISTANCE-ASSOCIATED MACROPHAGE PROTEIN 1*) (Fig 3A) [37] as well as *FRD3* (*FERRIC REDUCTASE DEFECTIVE 3*) and *NAS4* (*NICOTIANAMINE SYNTHASE 4*), two key genes involved in the transport of Fe through the xylem and phloem conducting tissues, respectively (Fig 3B) [38, 39]. It is noteworthy that S deficiency alone was sufficient to partly inhibit *NRAMP1* expression (Fig 3A). Altogether, these data suggested that the S deficiency signal might be integrated early in the transcriptional regulatory cascade governing the response to Fe deficiency in Arabidopsis.

**Fig 4:**
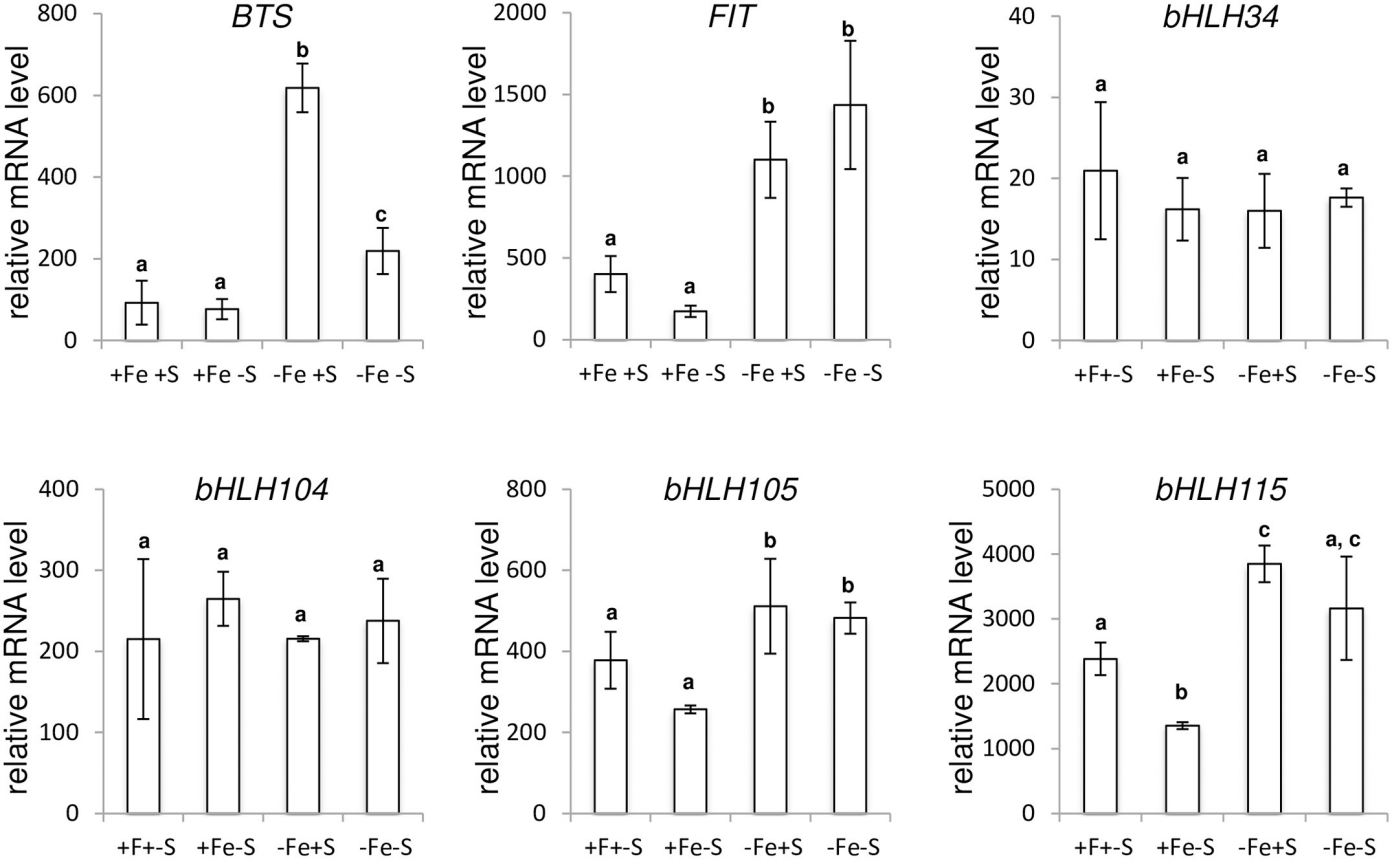
Expression analysis of genes involved in the early steps of the transcriptional regulatory network that controls *Arabidopsis thaliana* response to Fe deficiency. Quantitative RT-PCR analyses were carried out using cDNA synthesized from RNA extracted from roots of Arabidopsis plants grown for three weeks in presence of 25 μM Fe(III)-EDTA and transferred to four different media: control (+Fe +S), S deficiency (+Fe -S), Fe deficiency (-Fe +S) and Fe and S deficiencies (-Fe -S). Root samples were harvested five days after plants were transferred to the assayed media. The assayed genes encode a Fe-binding E3 ligase (*BTS*, *BRUTUS*), six bHLH (*FIT*/*bHLH29*, *bHLH34*, *bHLH104*, *bHLH105* and *bHLH115*) transcription factors. Means with the same letter are not significantly different according to one-way ANOVA followed by post-hoc Tukey test (*P*< 0.05). n = 3 biological repeats from one representative experiment. Each experiment was repeated three times.

**Fig 5:**
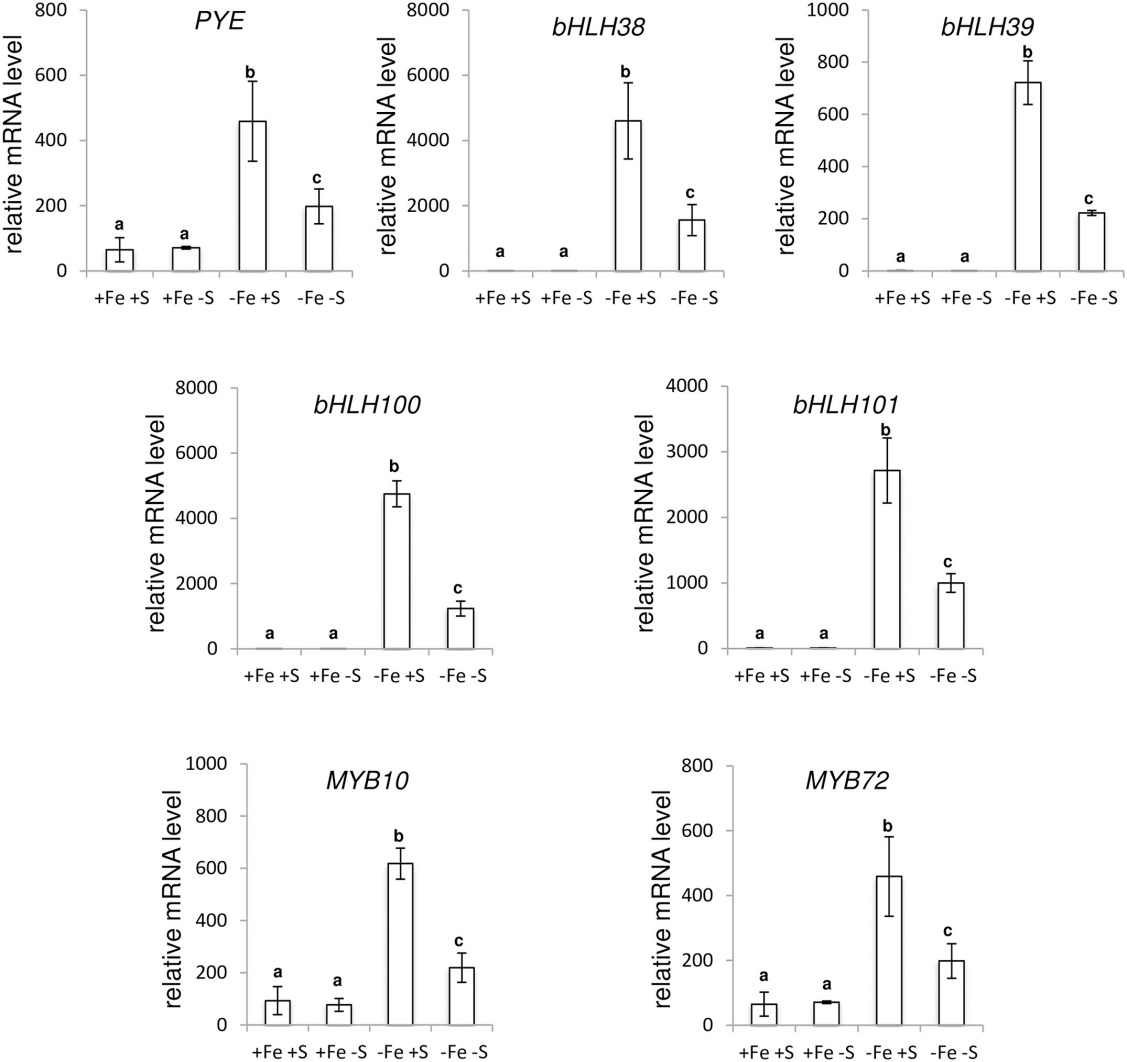
Expression analysis of genes involved in late steps of the transcriptional regulatory network that controls *Arabidopsis thaliana* response to Fe deficiency. Quantitative RT-PCR analyses were carried out using cDNA synthesized from RNA extracted from roots of Arabidopsis plants grown for three weeks in presence of 25 μM Fe(III)-EDTA and transferred to four different media: control (+Fe +S), S deficiency (+Fe -S), Fe deficiency (-Fe +S) and Fe and S deficiencies (-Fe -S). Root samples were harvested five days after plants were transferred to the assayed media. The assayed genes encode five bHLH (*PYE*/*bHLH47*: *POPEYE*, *bHLH38*, *bHLH39*, *bHLH100* and *bHLH101*) and two R2R3-MYB (*MYB10* and *MYB72*) transcription factors. Means with the same letter are not significantly different according to one-way ANOVA followed by post-hoc Tukey test (*P*< 0.05). n = 3 biological repeats from one representative experiment. Each experiment was repeated three times.

We thus examined the expression pattern of the main genes involved in the transcriptional regulatory cascade that govern the Arabidopsis response to Fe deficiency [21, 22, 40–46]. The main part of this regulatory network involves 10 bHLH transcription factors (TFs) belonging to four different clades, namely clade Ib (*i*.*e*. *bHLH38*, *bHLH39*, *bHLH100* and *bHLH101*), IIIa (FIT, FE-DEFICIENCY INDUCED TRANSCRIPTION FACTOR/bHLH29), IVb (PYE, *POPEYE*/*bHLH47*) and IVc (*bHLH34*, *bHLH104*, *bHLH105/ILR3* and *bHLH115*), and two homologous R2R3-MYBs (MYB10 and MYB72). Upstream this molecular network, *BTS* (*BRUTUS*) encodes a Fe-binding E3 ligase that mediates the degradation, through the 26S proteasome, of the clade IVc bHLH TFs [47]. We found that the induction of the expression of all the tested genes, but clade IVc bHLHs and *FIT*, in response to Fe deficiency was inhibited by a low S availability (Figs 4 and 5). This later observation was in agreement with the pattern of expression of all the structural genes involved in the maintenance of Fe homeostasis that were assayed (*i*.*e*. *IRT1*, *FRO2*, *PDR9*, *FRD3* and *NAS4*) and *NRAMP1* (Fig 3).

### The impact of sulphur scarcity on the chlorosis symptoms associated with iron deficiency do not involve SAL1-PAP in Arabidopsis

The way how S deficiency influences plant response to Fe deficiency is still to be discovered. Recent studies have evidenced that PAP (3'-phosphoadenosine 5'- phosphate), an intermediate compound into the assimilation of S, plays a central role in plant response to several environmental stresses (e.g. osmotic, cold, drought, high light, response to pathogens or cadmium excess) [30–32], in the chloroplast retrograde signalling pathway [24] and in the maintenance of Fe homeostasis [33]. PAP is formed from PAPS (3’-phosphoadenosine 5’-phosphosulfate) after the transfer of the sulphate group to target molecules (e.g. glucosinolates). PAP is an inhibitor of nuclear RNase (i.e. XRN2, XRN3 and XRN4, 5'-3' exonucleases) and affects the expression of several stress inducible genes. PAP accumulation is regulated by the phosphatase SAL1/FRY1 (FIERY1) in the chloroplast where it is catabolised into AMP and inorganic phosphate (Pi) [28]. In order to determine whether the inhibition of the Fe deficiency symptom when plants are subjected to both Fe and S deficiencies could be due to PAP, we have grown two loss-of-function mutant alleles of *SAL1*/*FRY1* (*i*.*e*. *fry1-6* and *alx8*) as well as the triple *xrn2 xrn3 xrn4* mutant in the presence or absence of Fe and/or S and compared them to wild type plants (S1 Fig). No differences in term of chlorosis (S1A Fig) and chlorophylls accumulation pattern (S1B Fig), in response to the four nutritional regimes that were assayed, were found between wild type plants and the three mutants. This result indicated that the cross talk occurring between Fe and S deficiency responses is most probably not involving PAP in Arabidopsis.

### Sulphur deficiency inhibits the iron deficiency induced accumulation of manganese in Arabidopsis plant tissues

**Fig 6:**
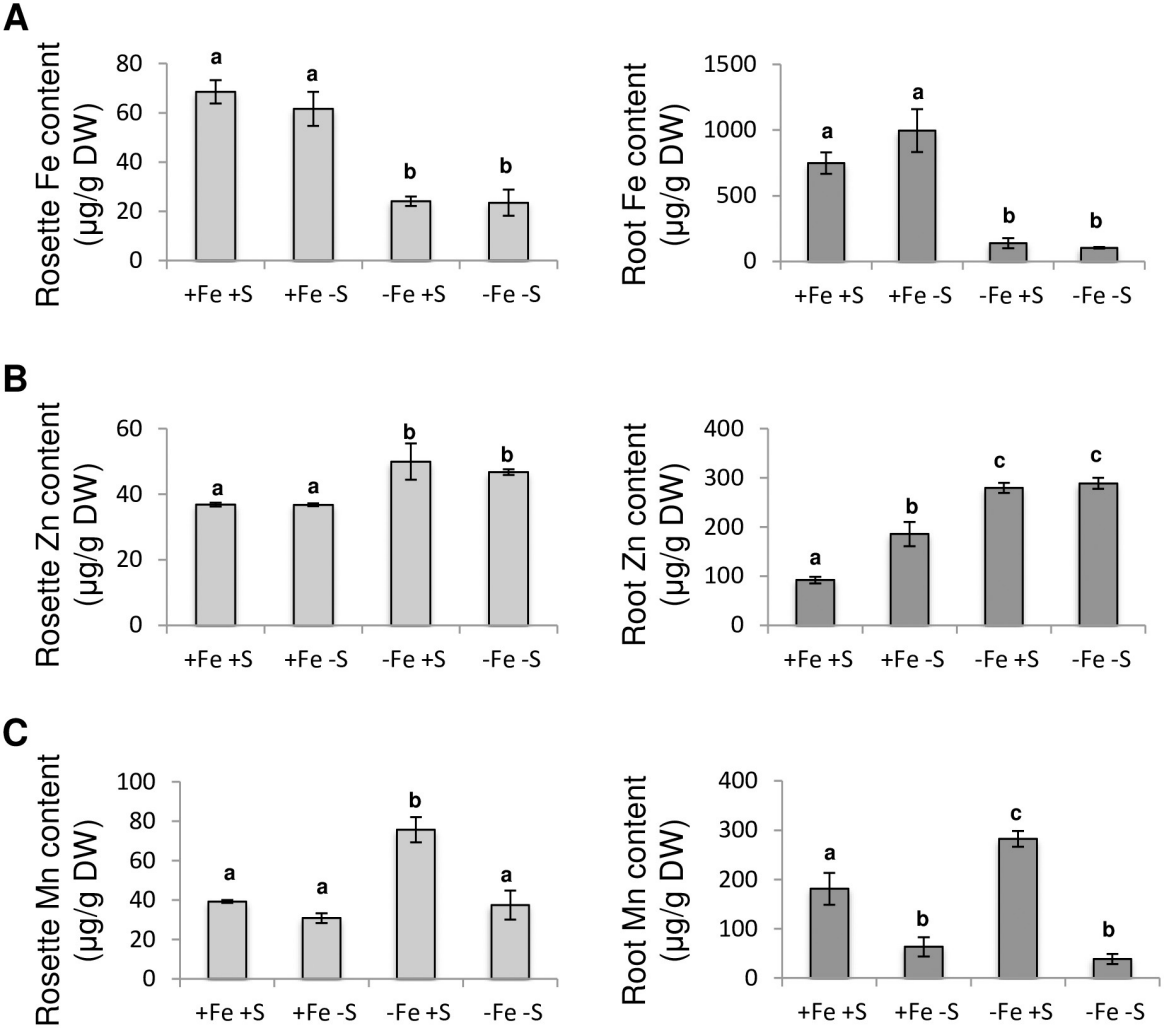
The increased accumulation of manganese in *Arabidopsis thaliana* plants in response to Fe deficiency is inhibited when S availability is scarce. **(A)** Fe, **(B)** Zn (zinc) and **(C)** Mn (manganese) content of rosette leaves and roots of Arabidopsis plants grown for three weeks in presence of 25 μM Fe(III)-EDTA and then transferred for 10 days in four different media: control (+Fe +S), S deficiency (+Fe -S), Fe deficiency (-Fe +S) and Fe and S deficiencies (-Fe -S). Means with the same letter are not significantly different according to one-way ANOVA followed by post-hoc Tukey test (*P*< 0.05). n = 3 biological repeats from one representative experiment. Each experiment was repeated three times.

Since leaf chlorophylls content is a proxy of the amount of Fe present in the tissues, we investigated whether Fe content in rosette leaves was increased when plants were facing Fe and S deficiencies when compared to plants facing Fe deficiency alone. We first observed that, under Fe deficiency, the amount of Fe present in the aerial tissues was lower than that of plants grown in control condition or under S deficiency (Fig 6A). We also found that S availability was not affecting the amount of Fe present in the aerial tissues, whether the plants were grown in presence or absence of Fe. Similar observations were made in roots.

We then investigated whether the observed phenotype could be due to some differential accumulation of zinc (Zn) and/or manganese (Mn) when plants are grown under Fe deficiency or under Fe and S deficiencies. This is because it is well established that in addition to transport Fe, IRT1 has the ability to transport Zn and Mn [48]. This is also because NRAMP1 is, in addition to being a low affinity Fe transporter, the major high affinity Mn transporter in Arabidopsis [49]. This hypothesis was supported by the expression patterns observed for *IRT1* and *NRAMP1* in Fe deficiency compared to Fe and S dual deficiency (Figs 1D and 3). We found that Zn accumulation was increased in both rosette leaves and roots when plants were grown under Fe deficiency and that S deficiency was not reverting this over-accumulation (Fig 6B). In contrast, we found that the increased accumulation of Mn in rosette leaves and roots associated with Fe deficiency was reverted when S availability was scarce (Fig 6C).

**Fig 7:**
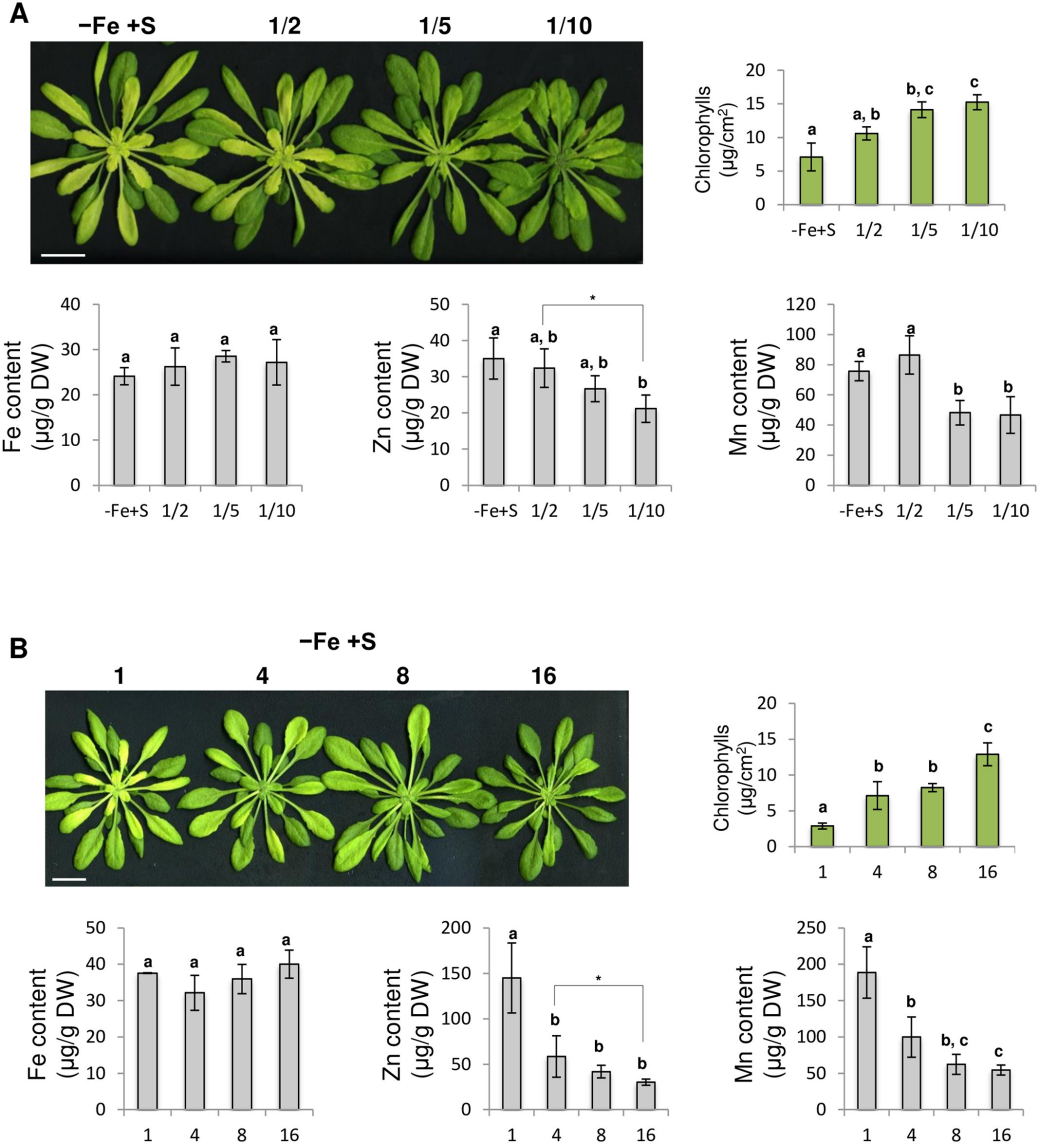
Manganese and zinc availability participates to the Fe deficiency induced chlorosis. **(A)** Effect of Mn and Zn availability on rosette leaves Fe deficiency induced chlorosis. Left panel: rosette phenotype of Arabidopsis plants grown for three weeks in presence of 25 μM Fe(III)-EDTA and then transferred for 10 days in Fe deficiency (-Fe +S) condition with various concentration of Mn and Zn. Mn and Zn concentration in Fe deficiency condition was 1/2, 1/5 and 1/10 of the initial -Fe +S medium. Bar = 1cm. Upper right panels: chlorophylls content of the youngest leaves of the rosettes presented upper left panel. Lower panels: Fe, Zn and Mn content of rosette leaves. **(B)** Effect of plant density on rosette leaves Fe deficiency induced chlorosis. Upper left panel: rosette phenotype of Arabidopsis plants grown for three weeks in presence of 25 μM Fe(III)-EDTA and then transferred for 10 days in Fe deficiency (-Fe +S) condition. Plants were grown at the density of 1, 4, 8 and 16 plants per 400 ml media. Bar = 1cm. Upper right panel: chlorophylls content of the youngest leaves of the rosettes presented upper left panel. Lower panels: Fe, Zn and Mn content of rosette leaves. **(A-B)** Means with the same letter are not significantly different according to one-way ANOVA followed by post-hoc Tukey test (*P*< 0.05). *: significant differences, *t*-test *p* < 0.05. n = 3 biological repeats from one representative experiment. Experiments displayed panel A and B were repeated three and two times, respectively.

These observations suggested that part of the Fe deficiency induced chlorosis symptoms might be due to an increased accumulation of Mn in plant tissues and additional divalent cations such as Zn. In order to test this hypothesis, plants were grown under Fe deficiency alone or under Fe deficiency with decreased concentration (i.e. 1/2, 1/5 and 1/10 of the initial concentration) of Mn, Zn or Mn and Zn. Fe deficiency induced chlorosis symptoms were similar when plants were grown under Fe deficiency or under Fe deficiency with decreased concentration of Mn or Zn (S2 Fig). In contrast, plants grown under Fe deficiency with reduced Mn and Zn concentrations in the growth medium displayed decreased Fe deficiency induced chlorosis symptoms that were positively correlated with the dilution factor (Fig 7A). Mn and Zn contents in rosette leaves were inversely correlated with chlorophylls content.

To investigate the effect of divalent cations accumulation on Fe deficiency induced chlorosis symptoms, Arabidopsis plants were then grown under Fe deficiency at different density (1, 4, 8 and 16 plants / 400 ml growth solution). This experiment showed that the Fe deficiency induced chlorosis was decreasing when the plant density was increasing, phenocopying the effect of S deprivation (Fig 7B). Mn and Zn contents in rosette leaves were also inversely correlated with chlorophylls content in this experiment.

Altogether these data suggest that part of the Fe deficiency induced chlorosis symptoms observed in Arabidopsis might be due to an accumulation of Mn, Zn and most probably other divalent cations. These data also suggest that the inhibition of the Fe deficiency induced chlorosis, when Arabidopsis plants are grown in the absence of S, could be due to an inhibition of, at least, Mn accumulation in plant tissues.

## Discussion

Iron (Fe) and sulphur (S) are two essential mineral nutrients for plant growth and development whose availability in soils affects crops productivity and the quality of their derived products [1]. Within the plant cell, Fe and S are tightly connected. The biogenesis of the Fe-S clusters, which are prosthetic groups essential for several proteins involved in key metabolic processes (e.g. photosynthesis, respiration), best exemplifies this interaction [2, 3, 5]. On a wider scale, Fe deficiency limits plant growth on one third of the cultivated land at the surface of the planet whereas, concomitantly, the surface of soils displaying S deficiency is increasing (because of a decrease of S release related to anthropogenic activities; [9]). Such observations indicate that the occurrence of Fe and S dual deficiency is likely to increase in the coming years and that sustaining plant growth and productivity in such limited environment, with lowering the use of exogenous fertilizers, will necessitate decrypting the mechanisms that govern plant Fe and S homeostasis interconnection. Pioneer work, carried out at the physiological and molecular levels, was initiated in order to document Fe and S interaction in different dicots (i.e. Arabidopsis, rapeseed, tomato) and mococots (i.e. barley, durum wheat, wheat) species [10–20]. Altogether, these studies have highlighted that modifications in the availability of one nutrient affects the homeostasis of the other, by notably modulating the expression and activity of several genes and proteins involved in these processes.

In order to get further insight into the interconnection between Fe and S homeostasis, we investigated, using Arabidopsis as model plant, how S deficiency signal was integrated into the transcriptional regulatory cascade controlling Fe deficiency responses [21, 22]. For this purpose, plants were submitted for 10 days to Fe, S or Fe and S deficiencies. We first observed, mostly in the youngest rosette leaves, that Fe deficiency induced chlorosis was less pronounced when plants were also subjected to S deprivation (Fig 1A and 1B). This observation was not in agreement with previous studies reporting that chlorophyll content of plant grown under both Fe and S deficiency was similar or lower to that of plant grown under Fe deprivation [10–13, 19]. However, similar observations to the ones described in this study were recently made in tomato [50]. In contrast, we found that S deficiency was not inducing any significant chlorosis, as described in wheat [13, 19] and tomato [50]. Nevertheless, S deficiency was reported to induce chlorosis in other plant species (i.e. barley, rapeseed, tomato). The use of specific physiological (anthocyanin accumulation) and molecular (i.e. *SULTR1;1*, *APR1*, *APK1* and *APK3* genes) markers confirmed that in our experiments the plants perceived the application of S deficiency (Fig 1C and 1E) and thus suggested that some species- or developmental stage-specific (i.e. seedlings vs mature plants, expanding vs mature leaves) mechanisms could be at play in the plant response to Fe, S or Fe and S deficiencies. Indeed, it could not be excluded that the growth parameters (e.g. day length, composition of the growth medium, stress application) may also influence these responses. However, as expected, we found a strong correlation between the chlorosis state of the plants and their photosynthetic capacities [10, 35] (Figs 1A, 1B and 2).

At the molecular level, the analysis of *IRT1*, *FRO2* and *PDR9* (three key genes involved in the Fe uptake machinery in Arabidopsis) mRNA abundance in roots unravelled common patterns of expression in response to Fe and S availability (Figs 1D and 3A). Their expression was strongly induced in response to Fe deficiency when compared to control condition and this induction was reduced when S availability was scarce (dual Fe and S deficiency). Such pattern of expression was previously reported for *IRT1* and *FRO2* in different species [10, 11, 16] even if this expression profile may fluctuate depending on the experimental conditions [15, 17]. Strikingly, although *IRT1*, *FRO2* and *PDR9* mRNA levels appeared lowered in S deficiency when compared to control condition, none of the observed differences was statistically significant. This is in contrast with the above-mentioned studies where *IRT1* and/or *FRO2* expression was either increased [10, 15] or decreased [11, 16, 17] in response to S deficiency when compared to control condition. Several reasons may explain these differences. In Arabidopsis, *IRT1* and *FRO2* expression was shown to decrease in response to S deficiency [11, 16, 17]. Such discrepancies might be due to the more stringent statistical test that was used in our study. Indeed, it cannot be excluded that it could also be the result of differences in the growth conditions that were used, in particular the composition of the growth medium. Indeed, one may expect variations that could be plant species dependent, even if similar discrepancies were reported in tomato [10, 15]. Our experiment revealed also that the expression pattern of *FRD3* and *NAS4* (two key genes involved in the transport of Fe through the xylem and phloem conducting tissues, respectively) was similar to that of *IRT1* (Fig 3B). This later result indicated that S deficiency modulates in a coordinated manner the expression of genes involved in Fe acquisition (*IRT1*, *FRO2* and *PDR9*) and transport (*FRD3* and *NAS4*), indicating that S deficiency inhibits Arabidopsis response to Fe shortage by modulating the expression of genes encoding regulatory proteins.

**Fig 8:**
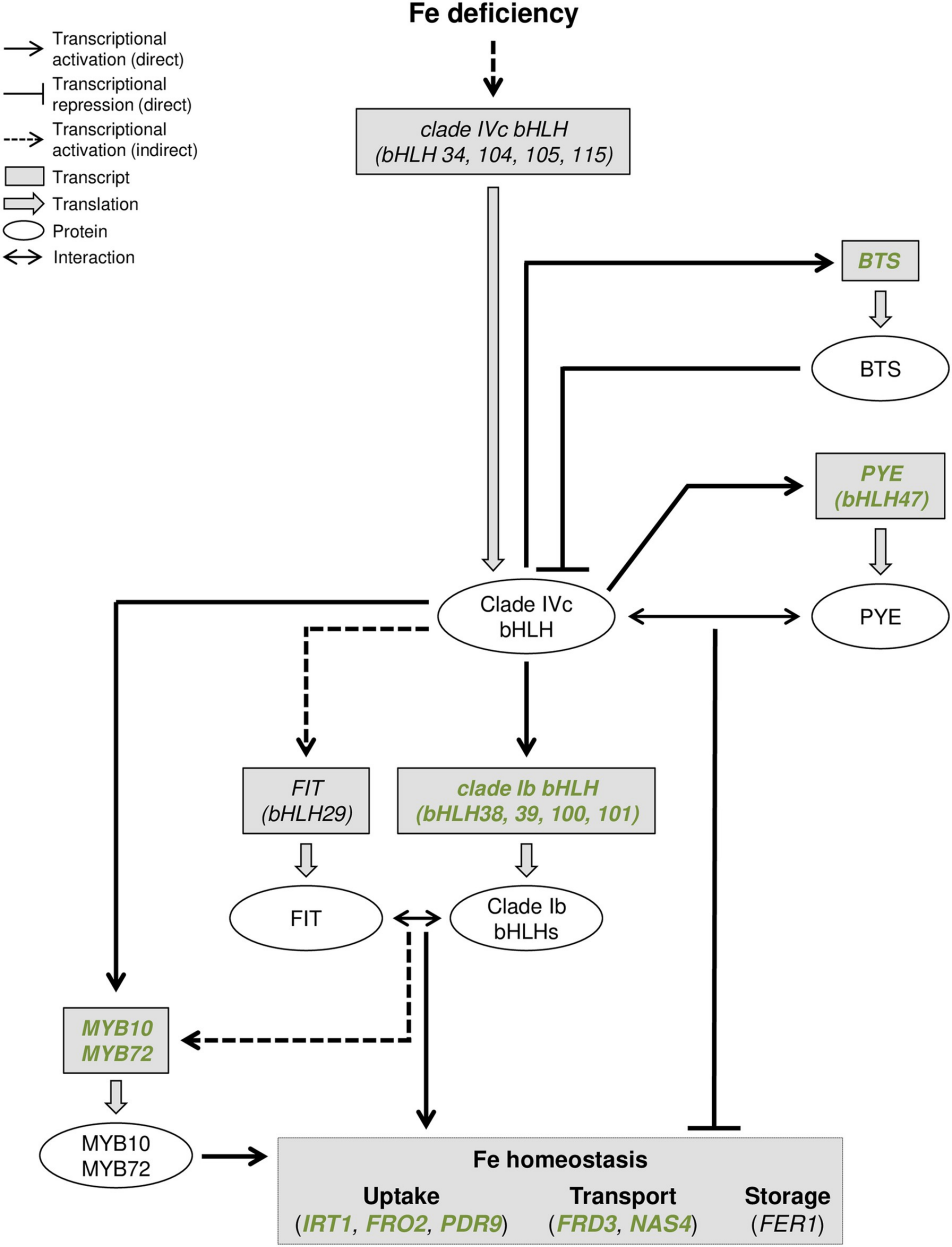
Schematic representation of the Fe deficiency response pathway in *Arabidopsis thaliana* and its connection with S shortage. Arabidopsis response to Fe shortage is essentially controlled at the transcriptional level and involves a complex regulatory network among which bHLH transcription factor (TF), from different clades [57] play a key role. The uppermost bHLH TF in this pathway belong to the clade IVc. The expression of these TF is induced in response to Fe deficiency. The stability of clade IVc encoded proteins is regulated by a Fe-binding E3 ligase, BTS (BRUTUS), whose expression is induced by clade IVc in response to Fe shortage. The expression of *PYE* (*POPEYE*, *bHLH47*), a transcriptional repressor, is also induced by clade IVc bHLH. PYE represses its target genes by interacting with bHLH105/ILR3 (IAA-LEUCINE RESISTANT3) [40, 46, 47]. In addition, clade IVc bHLH act as positive regulators [44, 58], in particular of clade Ib bHLH TF expression. Clade Ib bHLH act as transcriptional activators by forming heterodimers with another key bHLH TF, namely FIT (FE-DEFICIENCY INDUCED TRANSCRIPTION FACTOR, bHLH29). The expression of FIT is also induced in response to Fe shortage (indirect activation by the clade IVc bHLH TF). Downstream this pathway, two R2R3-MYB whose expression is dependent on FIT and clade IVc bHLH, MYB10 and MYB72, also act as transcriptional activators. The combined action of PYE, clade Ib bHLH TF, FIT, MYB10 and MYB72 contribute to maintain Fe homeostasis when Fe availability is scarce by regulating the expression of structural genes involved in Fe uptake, transport and storage. In green are the genes for which the Fe deficiency induced expression is reduced when both Fe and S deficiencies occurs.

This hypothesis relies on the fact that the regulatory network controlling the Arabidopsis response to Fe shortage is well described [21, 22, 44, 45, 58] (Fig 8). Upstream this network, four basic helix-loop-helix (clade IVc: bHLH34, bHLH104, bHLH105/ILR3 and bHLH115) transcription factors (TFs) directly activate the expression, when Fe availability is scarce, of a second set of bHLH TFs, namely *bHLH38*, *bHLH39*, *bHLH100*, *bHLH101* (calde Ib) and *bHLH47*/*PYE*. In parallel, clade IVc bHLHs also induce (indirect regulation) the expression of *bHLH29*/*FIT* [51]. FIT-dependent protein complexes are then formed, through heterodimerization with clade Ib bHLHs, leading to the direct activation of the expression of structural genes involved in the maintenance of Fe homeostasis such as *IRT1* and *FRO2*. Among the indirect targets of FIT, MYB10 and MYB72 (two R2R3-MYB TFs) play also an important role in maintaining plant growth under limited Fe availability notably by modulating the PDR9-dependent Fe uptake process [52]. In contrast, PYE acts as a repressor of genes especially involved in Fe transport such as *NAS4* [40, 53]. PYE activity relies on its interaction with ILR3 [40, 46, 47]. BTS, a Fe-binding E3 ligase, plays a key role in regulating this transcriptional regulatory cascade by modulating the stability of clade IVc bHLH TFs [40, 46, 47]. The study of the expression pattern of the different member of this regulatory network in response to Fe and/or S deficiency highlighted two types of mRNA accumulation profiles. First, we found that clade IVc bHLHs and *FIT* expression was unaffected by S deficiency, whether plants were subjected to Fe deficiency or not (Fig 5). The second pattern of expression was similar to that one described earlier for *IRT1* and concerned the downstream targets of clade IVc bHLHs (i.e. clade Ib bHLH and *PYE*) and *BTS* (Fig 4). It is noteworthy that the expression of *FIT*, *PYE*, *bHLH39* and *bHLH100* in response to Fe or Fe and S dual deficiency when compared to control condition was similar to what was reported in a previous study focusing on Arabidopsis [17]; however, in our study, and unlike what was previously reported, the inhibitory effect of S deficiency alone on the expression of these four genes was, like for *IRT1* and *FRO2*, not statistically significant. The inhibition by S deficiency of the Fe deficiency induced expression of the most downstream TFs in this regulatory network is intriguing (Fig 7). One would have expected such inhibition to occur upstream in this regulatory cascade, at the clade IVc bHLH and FIT levels. A hypothesis that could explain such observation would be that these TF play additional roles in the context of S deficiency. For instance, bHLH105/ILR3 was proposed to have a wider role in the plant response to various biotic and abiotic stresses [54]. Another hypothesis would be that clade IVc bHLHs and FIT regulation occurs at the posttranslational level, leading to their degradation via the 26S proteasome. In support of this latter hypothesis, some studies have highlighted the importance of this mechanism in the regulation of FIT [41, 55]. This would also involve a BTS independent mechanism for clade IVc bHLHs, as it is the case for MdbHLH104, an ortologue TF from apple [56]. Additional studies will be required to fully address this question in both Arabidopsis and other dicot crops. Altogether our data demonstrate that S deficiency integrates into the Arabidopsis Fe deficiency transcriptional regulatory network by regulating the expression of specific TFs (Fig 7) and most probably involves either additional transcriptional regulators competing with clade IVc bHLHs and FIT (repressors) or prosttranscriptional regulatory mechanisms affecting the stability of *BTS*, clade Ib bHLHs, *PYE*, *MYB10* and *MYB72*. Nevertheless, it is unlikely that this process involves the chloroplast SAL1-PAP retrograde signalling pathway (S1 Fig).

Because we observed that Fe deficiency induced chlorosis was less pronounced when plants were facing Fe and S dual deficiency, one could expect that Fe remobilisation into the aerial part would be increased. Measurements of Fe content in rosette leaves indicated that S deficiency was not influencing the amount of Fe measured in these tissues (Fig 6A). Similarly, we found that the amount of Fe present in roots was not dependent on S availability. Such pattern of Fe accumulation was in agreement with *FER1* expression (Fig 1D). Interestingly, the effect of S deficiency on the ability of dicot plants to accumulate Fe seems to be variable. For instance, in rapeseed, it was reported that S deficiency inhibits the accumulation of Fe in leaves and roots [10]. In tomato, the effect of S deficiency on the accumulation of Fe in leaves was reported to vary from a strong reduction (stronger than Fe deficiency itself) to no effect (as reported for Arabidopsis in this study) [11, 15, 18]. These observations indicate that the impact of S deficiency on Fe accumulation might be specific to the plant species, the developmental stage and/or the growth conditions. Another hypothesis to explain that Fe deficiency induced chlorosis was less pronounced when plants were facing Fe and S dual deficiency would be that the amount of other transition metals present in the plant tissues might fluctuate depending on S availability, and thus affect plant Fe deficiency induced chlorosis. In support of this hypothesis, it was recently demonstrated that excess of zinc (Zn), and to a lesser extent manganese (Mn), were phenocopying the Fe deficiency induced responses at the physiological and molecular levels [59]. In addition, it has been reported that Mn excess negatively impacts PSI activity but barely affects PSII [60]. The effect that we observed in the photosynthetic capacities indicates that the dual Fe and S deficiency reduced Mn toxicity since it improved QY (representative of total electron flux) but had no effect on Fv/Fm (that mesures PSII photochemical efficiency) [60] (Fig 2D and 2E). Another argument in support of this hypothesis is that the expression of *IRT1* (a low affinity Zn and Mn transporter; [48]) and *NRAMP1* (the major high affinity Mn transporter; [49]) followed, in our experiments, a similar expression pattern (i.e. lower induction in response to Fe deficiency when S availability is scarce) (Figs 1D and 3A). We first found that Zn and Mn content were significantly increased in rosette leaves of plants grown under Fe deficiency, when compared to plants grown in control condition, and that S deficiency alone was not affecting the amount of both metals, matching previous report observations [17, 59] (Fig 6B and 6C). In addition, we found that the amount of Mn present in rosette leaves of plants grown under Fe and S dual deficiency was lower than that of plants grown under Fe deficiency alone, and comparable to control condition (Fig 6C). Interestingly, we found that reducing the amount of Mn and Zn present in the growth medium of plants subjected to Fe deficiency had a positive effect on the Fe deficiency induced chlorosis (Fig 7A). Similarly, increasing the density of plants subjected to Fe deficiency was also having a positive effect on the Fe deficiency induced chlorosis that was accompanied by a decrease of Mn and Zn in rosette leaves (Fig 7B). Altogether, these results suggested that the effect of S deprivation on the Fe deficiency symptoms could be due to a differential accumulation of Mn and other divalent cations such as Zn in rosette leaves.

Data presented in this study show that S deficiency, by inhibiting part of the Arabidopsis Fe deficiency transcriptional regulatory network, negatively regulates the Fe uptake machinery that is induced in response to Fe deficiency (Fig 8). This process most probably limits the unspecific transport into the root and the plant body of potentially toxic divalent cations such as Mn and Zn by IRT1 and NRAMP1, thus limiting the deleterious effect of Fe deprivation. Indeed, it cannot be excluded that yet undiscovered transport mechanisms might also be at play. Whether S deficiency modulates the Fe deficiency transcriptional regulatory network in a similar manner in other dicots, in particular crops, will have to be investigated. This work emphasizes also the necessity to study the intricate connection that exists in plants between the homeostasis of macro- and micronutrients if one aims at sustaining plant growth and productivity in nutrients limited environment without the use of exogenous fertilizers. This is also of importance if one aims at improving nutritional and health quality of plant derived products by increasing specific micronutrient content, such as Fe, without accumulating undesired toxic metals.

## Materials and methods

### Plant material

*Arabidopsis thaliana* ecotype Columbia (Col-0) was used as wild type. The following mutant lines were used in this study: *fry1-6* (N520882), *alx8* (N66977) and the *xrn2 xrn3 xrn4* triple mutant [29].

### Growth conditions

Plants were hydroponically grown in a controlled growth chamber (23°C, 70% relative humidity, 200 μmol.m^-2^.s^-1^ light intensity) under short-day condition (8h light/16h dark photoperiod) as described elsewhere [36]. One plant per 100 ml Hoagland solution (S1 Table) density was used unless otherwise stated. Following 3 weeks of growth, in presence of 25 μM Fe(III)-EDTA, nutrient deficiencies were applied for 10 days. Nutrient deficiencies included: absence of iron (-Fe), absence of sulphur (-S), absence of iron and sulphur (-Fe -S). Following 5 days of nutrient deficiencies, roots were sampled and frozen in liquid nitrogen for subsequent genes expression analysis (qRT-PCR). This time point was chosen because it corresponds to the maximum of expression of genes involved in both Fe uptake and partitioning and the regulation of these processes [36]. Samples used for determining chlorophylls, anthocyanins and ions (i.e. iron: Fe; zinc: Zn; manganese: Mn) content as well as for western blot and chlorophyll fluorescence analysis were collected at the end of the experiment, after 10 days of nutrient deficiencies.

### Biochemical analyses

#### Chlorophylls content

Chlorophylls from 5 leaf discs (diameter: 0.35 cm) were extracted in 1 ml 100% acetone in the dark under agitation. The absorbance (A) at 661.8 and 644.8 nm was then measured. Total chlorophylls content was assessed using the following equations: Chl a + Chl b = 7.05*A_661.6_ + 18.09*A_644.8_ and expressed as μg/cm^2^ [61].

#### Anthocyanins content

Anthocyanins from 50 mg of leaves (FW: fresh weight) homogenized in 1 ml acidic methanol (1% HCl, w/v) were extracted in the dark at 4°C (moderate shaking, overnight). Following centrifugation (5 min, 14.000 rpm, room temperature), the absorbance of the supernatant (A) at 530 and 657 nm was measured. Total anthocyanins content was assessed using the following equations: Anthocyanins = (A_530_ − (0.25*A_657_)) and expressed as A_Anthocyanins_ /mg FW.

#### Iron, manganese and zinc determination

About 15 mg (DW: dry weight) of ground sample (rosette leaves or roots) were mixed with 750 μl nitric oxide (65%) and 250 μl hydrogen peroxide 30% before homogenization. Samples were left over night at room temperature and then mineralized 7h at 84°C. Once mineralized, the nitric oxide proportion present in the samples was adjusted to 5 to 10% of the final volume by adding ultrapure water. Minerals content present in the samples was then measured by microwave plasma atomic emission spectroscopy (MP-AES, Agilent).

### Chlorophyll fluorescence

A kinetic imaging fluorometer (FluorCam FC 800-O, Photon Systems Instruments, Czech Republic) was used to capture chlorophyll fluorescence images and to estimate the maximal quantum yield [F_v_/F_m_ = (F_m_−F_o_)/F_m_] and the effective quantum yield of the photosystem II (φ_PSII_; under 200 μmol·m^−2^·s^−1^ actinic light). Plants were first dark-adapted for 30 min and saturating flashes (0.8 s, 5000 μmol·m^−2^·s^−1^) were then shot to measure the maximum fluorescence. Measurements were performed at room temperature.

### Gene expression analysis (qRT-PCR)

Total RNAs was extracted using the Tri-Reagent (Molecular Research Center) method. Briefly, each sample was homogenized in 1 ml Tri-Reagent solution mixed with 160 μl of chloroform:isoamyl alcohol (24:1). Following centrifugation (10 min, 13.000 rpm, 4°C) total RNAs present in the aqueous phase were precipitated by the addition of 400 μl of isopropanol followed by another centrifugation. Pellets were then washed twice with ethanol 70% and dried prior resuspension in RNAse-free water. For each sample, 1 μg of total RNA treated with DNase was reverse transcribed into cDNA using the RevertAid kit (Thermo scientific). Quantitative PCR analyses were carried out using a LightCycler^®^ 480 (Roche) and the LC480-SYBR-Green master I reaction mix (Roche). *PP2AA3* (*PROTEIN PHOSPHATASE 2A SUBUNIT A3*) was used as reference gene [62]. Expression levels were calculated using the comparative threshold cycle method. All the primers used are described S2 Table.

### Western blot analysis

Total proteins were extracted from 100 mg of samples (rosette leaves) grinded in liquid nitrogen and homogenized in 250 μl of 1x extraction buffer (100 mM Tris-HCl pH 8, 1% LDS, 25 mM EDTA, 5% ß-mercaptoethanol (v/v), 2 mM PMSF). Following two consecutive centrifugations the supernatant was collected and stored on ice prior use. Protein content was determined using the Bradford assay [63]. Proteins were then separated by 12% SDS-PAGE (Laemmli system) and transferred to a PVDF membrane. Membranes were blocked for overnight in TBS buffer (50 mM Tris pH 7.5, 150 mM NaCl, 0.1% Tween-20, 1% BSA) and then incubated 1h with primary antibody and then 1 h with secondary antibody, both diluted in TBS containing 1% BSA. Dilutions of primary antibodies applied were: rabbit anti-PsaD 1:10.000 [64], rabbit anti-b6/f 1:5.000 [64], rabbit anti-PsbA 1:20.000 (Agrisera, AS05084) and goat anti-rabbit HRP conjugated (Promega, W4011) 1:10.000. Immunodetection (LAS-3000 Imaging System, Fuji) was performed using the Luminata Forte (Deutscher) solution.

### Studied Arabidopsis gene IDs

*APK1/AKN1*, At2g14750; *APK3*, At3g03900; *APR1*/*PRH19*, At4g04610; *bHLH29/FIT*, At2g28160; *bHLH34*, At3g23210; *bHLH38*, At3g56970; *bHLH39*, At3g56980; *bHLH47/PYE*, At3g47640; *bHLH100*, At2g41240; *bHLH101*, At5g04150; *bHLH104*, At4g14410; *bHLH105/ILR3*, At5g54680; *bHLH115*, At1g51070; *BTS*, At3g18290; *FER1/AtFER1*, At5g01600; *FRD3*, At3g08040; *FRO2*, At1g01580; *FRY1/SAL1/ALX8/HOS2/RON1/RON1/SUPO1*, At5g63980; *IRT1*, At4g19690; *MYB10*, At3g12820; *MYB72*, At1g56160; *NAS4*, At1g56430; *NRAMP1*, At1g80830; *PDR9/ABCG37*, At3g53480; *PP2AA3*, At1g13320; *SULTR1;1*, At4g08620; *XRN2*, At5g42540; *XRN3*, At1g75660; *XRN4*, At1g54490.

## Supporting information

S1 Fig SAL1-PAP retrograde signal is not involved in the attenuation of *Arabidopsis thaliana* Fe deficiency induced chlorosis when S availability is scarce. **(A)** Rosette phenotype of Arabidopsis plants grown for three weeks in presence of 25 μM Fe(III)-EDTA and then transferred for 10 days in four different media: control (+Fe +S), S deficiency (+Fe -S), Fe deficiency (-Fe +S) and Fe and S deficiencies (-Fe -S). Wild type (WT) plant and three mutants were analysed, namely two loss-of-function alleles of the phosphatase SAL1/FRY1 (*fry1-6* and *alx8*) and a triple mutant of the nuclear 5'-3' exoribonucleases XRN2, XRN3 and XRN4 (*xrn2 xrn3 xrn4*) whose activity is regulated by SAL1/FRY1. Bar = 1 cm. **(B)** Chlorophylls content of the youngest leaves of the rosette presented panel A. Means with the same letter are not significantly different according to one-way ANOVA followed by post-hoc Tukey test (*P*< 0.05). n = 3 biological repeats from one representative experiment. Each experiment was repeated three times.
(PDF)

S2 Fig Effect of manganese and zinc deficiency on rosette leaves Fe deficiency induced chlorosis. Arabidopsis plants were grown for three weeks in presence of 25 μM Fe(III)-EDTA and then transferred for 10 days in Fe deficiency (-Fe +S) condition alone or with a concentration of Mn or Zn that was 1/10 of the initial -Fe +S medium. Bar = 1cm.
(PDF)

S1 Table Composition of the Hoagland solutions used in this study. (PDF)

S2 Table Primers used in this study. (PDF)
