# Supplementary figures and images for: Sulphur availability modulates Arabidopsis thaliana responses to iron deficiency
Source: PLoS One. 2020 Aug 20;15(8):e0237998. doi: 10.1371/journal.pone.0237998 (PMC7440645; doi:10.1371/journal.pone.0237998)

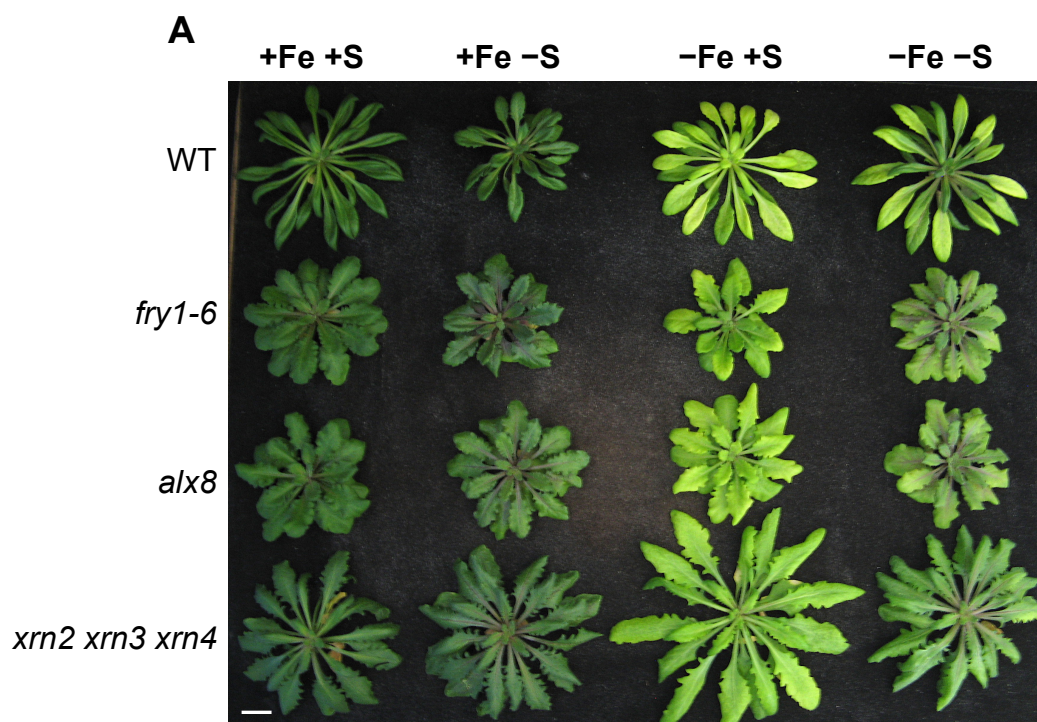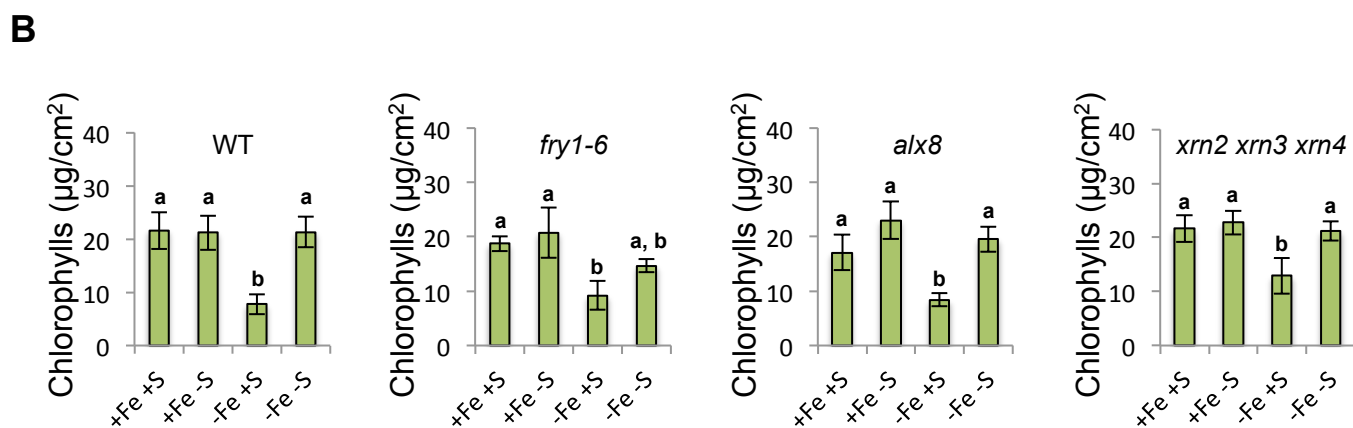

**S1 Figure**

Supplement: S1 Fig — (A) Rosette phenotype of Arabidopsis plants grown for three weeks in presence of 25 μM Fe(III)-EDTA and then transferred for 10 days in four different media: control (+Fe +S), S deficiency (+Fe -S), Fe deficiency (-Fe +S) and Fe and S deficiencies (-Fe -S). Wild type (WT) plant and three mutants were analysed, namely two loss-of-function alleles of the phosphatase SAL1/FRY1 (fry1-6 and alx8) and a triple mutant of the nuclear 5'-3' exoribonucleases XRN2, XRN3 and XRN4 (xrn2 xrn3 xrn4) whose activity is regulated by SAL1/FRY1. Bar = 1 cm. (B) Chlorophylls content of the youngest leaves of the rosette presented panel A. Means with the same letter are not significantly different according to one-way ANOVA followed by post-hoc Tukey test (P< 0.05). n = 3 biological repeats from one representative experiment. Each experiment was repeated three times. (PDF) [file pone.0237998.s001.pdf]

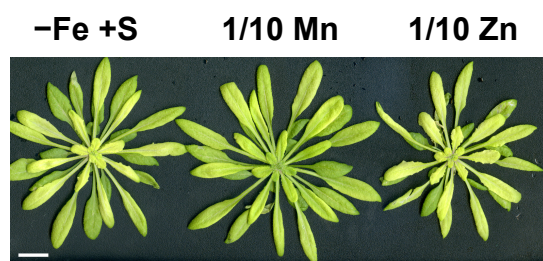

**S2 Figure**

Supplement: S2 Fig — Arabidopsis plants were grown for three weeks in presence of 25 μM Fe(III)-EDTA and then transferred for 10 days in Fe deficiency (-Fe +S) condition alone or with a concentration of Mn or Zn that was 1/10 of the initial -Fe +S medium. Bar = 1cm. (PDF) [file pone.0237998.s002.pdf]
