# Supplementary material for: Sulphur availability modulates Arabidopsis thaliana responses to iron deficiency
Source: PLoS One. 2020 Aug 20;15(8):e0237998. doi: 10.1371/journal.pone.0237998 (PMC7440645; doi:10.1371/journal.pone.0237998)
Supplement: S1 Table — (PDF) [file pone.0237998.s003.pdf]

S1 **Table:** Hogland solutions (g per liter)

		+Fe +S	+Fe -S	-Fe +S	-Fe -S
**Solution #1** Macronutrients 100X stock	KH_2PO_4	13	13	13	13
	KNO_3	50	50	50	50
	$MgSO_4$	24	0	24	0
	$MgCl_2$, 6 H_20	0	20.3	0	20.3
**Solution #2** Micronutrients 1000X stock	H_3BO_3	3.09	3.09	3.09	3.09
	CoCl, H_20	0.012	0.012	0.012	0.012
	$CuSO_4$, H_2O	0.048	0	0.048	0
	$CuCl_2$, 2 H_2O	0	0.034	0	0.034
	$ZnSO_4$, 7 H_2O	0.34	0	0.34	0
	$ZnCl_2$, 7 H_2O	0	0.163	0	0.163
	KI	0.41	0.41	0.41	0.41
	$MnSO_4$	0.84	0	0.84	0
	$MnCl_2$, 4 H_20	0	0.99	0	0.99
	MoNa, 2 H_20	0.072	0.072	0.072	0.072
**Solution #3**100X Stock	$CaNO_3$, 4 H_20	118	118	118	118
**Solution #4** 1000X Stock	Fe-EDTA	0.0092	0.0092	0	0
