# Supplementary material for: Sulphur availability modulates Arabidopsis thaliana responses to iron deficiency
Source: PLoS One. 2020 Aug 20;15(8):e0237998. doi: 10.1371/journal.pone.0237998 (PMC7440645; doi:10.1371/journal.pone.0237998)
Supplement: S2 Table — (PDF) [file pone.0237998.s004.pdf]

S2 Table: Primers used i n this study

Name	Sequence	Gene ID
bHLH29/FIT-Q1	CAGTCACAAGCGAAGAAACTCA	At2g28160
bHLH29/FIT-Q2	CTTGTAAAGAGATGGAGCAACACC	At2g28160
bHLH34-Q1	TCGTCATCTGTTGGAGCTGT	At3g23210
bHLH34-Q2	GTTTCTCGCGACAGGCTTTG	At3g23210
bHLH38-Q1	AGGAGAGAGGCTCTTCTACACTT	At3g56970
bHLH38-Q2	TGAGAACTAGTGGATAAACACACCA	At3g56970
bHLH39-Q1	GACGGTTTCTCGAAGCTTG	At3g56980
bHLH39-Q2	GGTGGCTGCTTAACGTAACAT	At3g56980
bHLH47/PYE-Q1	CAGGACTTCCCATTTTCCAA	At3g47640
bHLH47/PYE-Q2	CTTGTGTCTGGGGATCAGGT	At3g47640
bHLH100-Q1	CTCCCACCAATCAAACGAAGAAG	At2g41240
bHLH100-Q2	TGTTTTGGTCGGTGTAAACGAG	At2g41240
bHLH101-Q1	AAGAAGATCGAGGAGCGGTG	At5g04150
bHLH101-Q2	AGAGGCAAGAGAGCACGAAG	At5g04150
bHLH104-Q1	CCAGCTGCATTTAACCACAACA	At4g14410
bHLH104-Q2	TTAAGCAGCAGGAGGCCTGAG	At4g14410
bHLH105-Q1	GCAACCTATTGGTGTTTCTTCTAACTC	At5g54680
bHLH105-Q2	CCAGGTTCTTTGCTAGCTTCTGA	At5g54680
bHLH115-Q1	TCAAGCAAGAGATGAAGCGC	At1g51070
bHLH115-Q2	GACAAGCTTGCTTCCAGGAG	At1g51070
BTS-Q1	ACCATGTCGATCTCCGGCTG	At3g18290
BTS-Q2	CAAGAGAATATGTTTGCGCTACATT	At3g18290
FER1-Q1	TCGTTGAGAGTGAATTTCTGG	At5g01600
FER1-Q2	ACCCCAACATTGGTCATCTG	At5g01600
FRD3-Q1	CAACCTCCAGCTCCGGATAC	At3g08040
FRD3-Q2	GTGTGCTGGTGATAACATTGG	At3g08040
FRO2-Q1	GCGACTTGTAGTGCGGCTATG	At1g01580
FR02-Q2	CGTTGCACGAGCGATTCTTG	At1g01580
IRT1-Q1	CGGTTGGACTTCTAAATGC	At4g19690
IRT1-Q2	CGATAATCGACATTCCACCG	At4g19690
MYB10-Q1	GGGGAAATCTTGGTGGAGCA	At3g12820
MYB10-Q2	AGGAGGAACCTGGCTATCGT	At3g12820
MYB72-Q1	TCGAGAGGTAACCAAATCGCA	At1g56160
MYB72-Q2	CAGCTGTCTCCTCAAGTCGG	At1g56160
NAS4-Q1	GGCTTCGACGTTGTGTTCTT	At1g56430
NAS4-Q2	AGCAAAGCACCAGGAGACAT	At1g56430
PDR9-Q1	GCGAAACTCAGAGCTTGTGA	At3g53480
PDR9-Q2	AGTGCGCCGAAGATCAAAGA	At3g53480
PP2A3-Q1	TAACGTGGCCAAAATGATGC	At1g13320
PP2A3-Q2	GTTCTCCACAACCGCTTGGT	At1g13320
SULTR1;1-Q1	TTGCTCAGCCACTTCCGTAC	At4g08620
SULTR1;1-Q2	CTCAAGAGCCTCGAGAAGC	At4g08620
APR1-Q1	CATTGGAGCCAAAAGTTTCGC	At4g04610
APR1-Q2	TCCTCAATCTCAACCACATCAAC	At4g04610
APK1-Q1	CCTTACGAGCCACCATTGAACTG	At2g14750
APK1-Q2	GCCATTTCGATAGGAGAAGTTCCT	At2g14750
APK3-Q1	GTTGAAAGAGAAAGAGGGAGAGTGTC	At3g03900

**APK3-Q2**	AGAGATCACTTCCTCAGCCATAGC	At3g03900
**NRAMP1-Q1**	CGGAACTTATGCTGGACAAT	At1g80830
**NRAMP1-Q2**	AGAAGAGGAACCAACGCAAA	At1g80830
